# Experience with chemotherapy for postoperative metastases of adenosquamous carcinoma of the esophagogastric junction and pathological study of its development

**DOI:** 10.1093/jscr/rjae440

**Published:** 2024-07-03

**Authors:** Kazuhito Mita, Hideaki Oda, Mayu Shimaguchi, Michitaka Kouno, Naoyuki Toyota, Minoru Hatano, Tsuyoshi Toyota, Junichi Sasaki

**Affiliations:** Department of Surgery, Tsudanuma Central General Hospital, 1-9-17 Yatsu, Narashino, Chiba 275-0026, Japan; Department of Diagnostic Pathology, Tsudanuma Central General Hospital, 1-9-17 Yatsu, Narashino, Chiba 275-0026, Japan; Department of Surgery, Tsudanuma Central General Hospital, 1-9-17 Yatsu, Narashino, Chiba 275-0026, Japan; Department of Surgery, Tsudanuma Central General Hospital, 1-9-17 Yatsu, Narashino, Chiba 275-0026, Japan; Department of Surgery, Tsudanuma Central General Hospital, 1-9-17 Yatsu, Narashino, Chiba 275-0026, Japan; Department of Surgery, Tsudanuma Central General Hospital, 1-9-17 Yatsu, Narashino, Chiba 275-0026, Japan; Department of Surgery, Tsudanuma Central General Hospital, 1-9-17 Yatsu, Narashino, Chiba 275-0026, Japan; Department of Surgery, Tsudanuma Central General Hospital, 1-9-17 Yatsu, Narashino, Chiba 275-0026, Japan

**Keywords:** adenosquamous carcinoma, esophagogastric junction, S-1

## Abstract

We report here a case of postoperative recurrent adenosquamous carcinoma (ASC) of the esophagogastric junction (EGJ) treated with S-1 therapy. A 79-year-old woman was diagnosed with carcinoma of the EGJ. Thoracoscopic subtotal esophagectomy was performed, and pathological examination revealed advanced ASC with lymph node metastasis. Five months after surgery, multiple lung metastases and multiple lymph node metastases were observed, and the patient was treated with S-1 monotherapy, which showed partial response and may be effective for advanced ASC of the EGJ. On the other hand, immunohistological analysis of the tumors showed a relatively wide range of areas that could differentiate into both adenocarcinoma and squamous cell carcinoma, suggesting that tumor cells with multidifferentiation potential, or at least the ability to differentiate into both adeno-epithelial and squamous epithelial cells, were the likely source of the tumors.

## Introduction

Adenosquamous carcinoma (ASC) of the esophagus or stomach is reported incidence rates of 0.37–1% among all esophageal cancers in the West and 0.6–1% in Japan [1, 2] and reported to be less than 1% of all gastric cancers in Europe and the United States, and 0.14–1.3% in Japan [1, 2]. We report a case of ASC of the esophagogastric junction (EGJ) treated with surgery and chemotherapy for metastases and discuss its origin and chemotherapy.

## Case report

**Figure 1:**
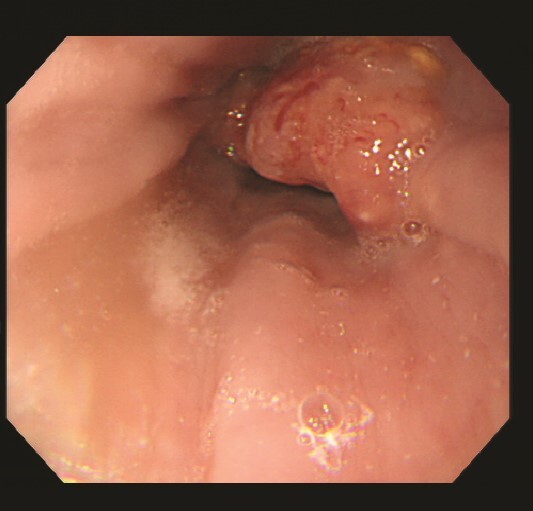
Upper gastrointestinal endoscopy revealed a type 3 esophagogastric junction tumor that had invaded the esophagus by 2 cm. Endoscopically, the neck of the tumor was located at the junction, and together with the histopathologic results, a diagnosis of Siewert type 2 esophagogastric junction carcinoma was made. Initially, a squamous cell carcinoma component and an adenocarcinoma component were observed, and the patient was suspected of having basal cell carcinoma.

The patient is a 79-year-old woman, complaining of dysphagia, who came to our department after a CT scan with an abnormality in the lower esophagus. Type 3 tumor was found at the EGJ (Fig. 1). Biopsy results showed that the surface of the tumor was covered with non-neoplastic squamous epithelium, and the area directly below the squamous epithelium was occupied by a squamous cell carcinoma component and an adenocarcinoma-like component. Based on TNM classification, it was cT3 cN2 M0 cStageIII. The patient underwent thoracoscopic subtotal esophagectomy and 2-region dissection with a diagnosis of advanced EGJ cancer. The patient was discharged from the hospital 17 days after surgery.

**Figure 2:**
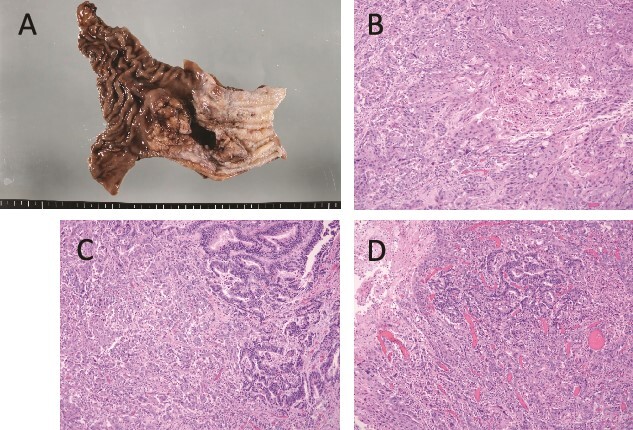
Macroscopic and microscopic findings. (A) Macroscopic findings of the resected specimen. A type 3 tumor of about 5 cm with a tumor center is seen at the esophagogastric junction. (B) Histological findings, showing a squamous cell carcinoma area. The tumor was found mainly in the superficial layer of the tumor and not in the deeper layers. (C) Boundary between well-differentiated (left side) and poorly differentiated (right side) adenocarcinoma. The tumor was found to be poorly differentiated adenocarcinoma-like mainly in the middle to deep layers of the tumor center. The superficial part of the tumor was transformed into a well-differentiated adenocarcinoma. (D) The boundary area between the three cancer components is shown. The squamous cell carcinoma component and the well-differentiated adenocarcinoma component are bordered by the superficial portion and the poorly differentiated adenocarcinoma-like portion in the deeper portion, respectively.

**Figure 3:**
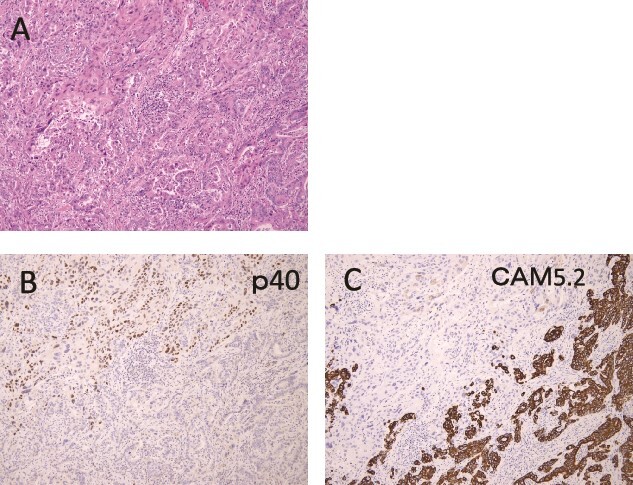
Immunohistochemical findings are shown. (A) Hematoxylin and Eosin staining specimen with histological evidence of adenocarcinoma (lower half) and squamous cell carcinoma (upper half) components. (B) The squamous cell carcinoma component showed p40-positive findings. (C) On the other hand, CAM5.2 positive findings were observed in the adenocarcinoma component.

**Figure 4:**
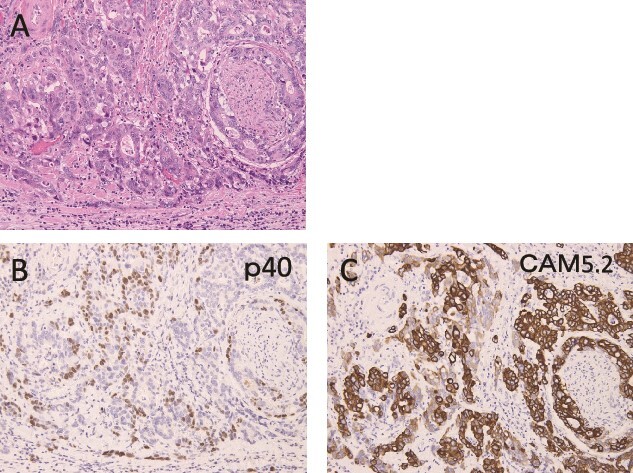
Immunohistochemical findings are shown. (A) Hematoxylin and eosin staining shows areas of moderately or poorly differentiated adenocarcinoma. (B, C) p40-positive cells were observed, mixed with CAM5.2-positive cells, and only one of them was positive.

Pathologically, it was a type 3 tumor with a tumor center at the EGJ (Fig. 2A), and histologically, the diagnosis was ASC with adenocarcinoma (AC) and squamous cell carcinoma (SCC) portions. Therefore, it was pT3 pN3 M0 pStage VIa. Microscopically, the AC area was relatively wide from superficial to deep, occupying about 70% of the maximum circumferential surface. Well differentiated adenocarcinomas were found in the superficial layers, and moderately or poorly differentiated adenocarcinomas in the middle to deeper layers. SCC areas were found mainly around the ulcer in the superficial layers of the tumor, and were also scattered in the middle layers, but no clear squamous cell carcinoma was seen in the deeper layers (Fig. 2B–D). The boundary between this AC area and the SCC area was indistinct. The adenocarcinoma component accounted for the majority of lymph node metastases. Immunohistochemistry was performed, using p40, the most reliable marker for SCC, and CAM5.2, a non-squamous epithelial marker. The histological diagnosis was confirmed by p40-positive images in the area thought to be SCC and CAM5.2-positive images in the area thought to be AC (Fig. 3). This immunohistochemical search revealed scattered p40-positive cells in areas that appeared to be moderately or poorly differentiated adenocarcinomas, which were CAM5.2 negative (Fig. 4).

**Figure 5:**
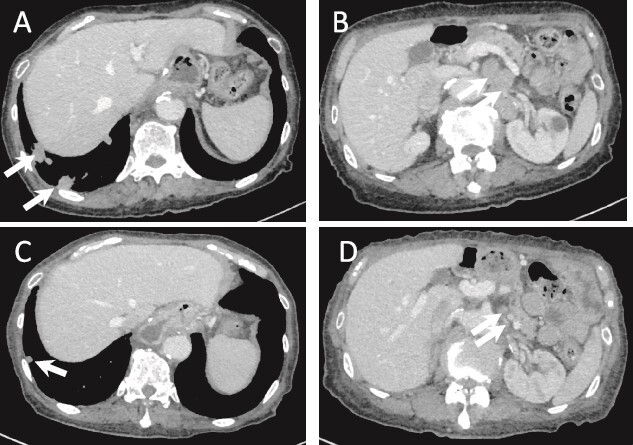
(a) CT image 5 months after surgery. Multiple lung metastases are seen (arrows). (B) Intra-abdominal lymph node metastasis is shown (arrows). (C, D) Lung and lymph node metastases after one cycle of chemotherapy show PR (arrows).

Five months after surgery, a CT scan showed multiple lung and lymph node metastases. CT scan after one cycle of S-1 monotherapy showed partial response (PR) (Fig. 5). Ten months after surgery, the patient became progressive disease (PD). S-1 monotherapy was continued until 1 year and 2 months postoperatively, but treatment was terminated due to deterioration of performance status (PS 3), and the patient died at 1 year and 5 months postoperatively.

## Discussion

There are five developmental hypotheses for ASC of the stomach [3, 4]. [1] squamous transformation of adenocarcinoma, [2] carcinogenesis of ectopic squamous epithelium, [3] carcinogenesis of the metaplastic non-neoplastic squamous cells, [4] differentiation of multipotent undifferentiated cancer cells into both glandular and squamous cells, and [5] collision of adenocarcinoma and squamous cell carcinoma at the same time.

On the other hand, there are several hypotheses for the development of ASC of the esophagus [1] originating from the esophageal intrinsic gland or conduit epithelium, [2] ASC originating from the mucosa and first developing into SCC, followed by differentiation of the adenocellular cells into adenosquamous cells, [3] simultaneous collision of AC and SCC [1, 5, 6].

In this case, histopathological search showed that the tumor center was located at the EGJ, and the AC and SCC portions were clearly demonstrated. In addition, the AC portion accounts for 70% and the SCC portion for 30%, and the immunohistochemical search confirmed both, so the diagnosis of ASC of the EGJ is reasonable. On the other hand, immunohistological search revealed that p40-positive cells, a marker for squamous cell carcinoma, were also scattered in the adenocarcinoma-like areas. The results of our search in this study show a relatively wide range of areas that can differentiate into both AC and SCC. Therefore, tumor cells that possess multidifferentiation potential, or at least the ability to differentiate into both glandular and squamous cells, are considered to be their developmental matrix.

Regarding chemotherapy for advanced ASC, Chen argued that the prognosis was similar to that of esophageal squamous cell carcinoma [7]. Evans *et al*. reported that esophageal ASC is more metastatic than AC or SCC [8]. Yendamuri’s analysis shows that esophageal ASC is diagnosed at a more advanced stage than AC and has a poorer prognosis than AC [6]. Gamboa *et al*. [9] reported that the response of esophageal ASC to radiation chemotherapy is similar to that of AC.

As for gastric ASC, Ebi *et al*. reported recurrence-free survival after postoperative S-1 monotherapy in patients with positive cancer cells on intraperitoneal washing cytology (CY1) [3]. Hirano *et al*. [10] reported recurrence-free survival in patients with gastric ASC with peritoneal dissemination treated with postoperative S-1 plus paclitaxel (PTX). Yugang *et al*. [11] suggested that gastric ASC has a worse prognosis than AC. Chen *et al*. reported the use of S-1 plus oxaliplatin (SOX), fluorouracil plus folinic acid and irinotecan (FOLFIRI), and fluorouracil plus leucovorin and oxaliplatin (FOLFOX) therapy for gastric ASC. They also reported that it is diagnosed at a more advanced stage, has a poorer prognosis, and lymph node metastasis of the AC component may determine the prognosis [12].

We used S-1 monotherapy for multiple metastases because (i) the patient refused the intravenous chemotherapy regimen, (ii) the treatment of gastric cancer was followed because the lymph node metastasis was mainly adenocarcinoma component, (iii) S-1 monotherapy has been reported to be effective in elderly patients with esophageal cancer [13], and (iv) in elderly patients with gastric cancer, S-1 monotherapy showed no clear prognostic difference compared to S-1 plus CDDP [14]. Since a PR was observed with S-1 monotherapy, we believe that S-1 monotherapy can be expected to have a certain therapeutic effect in the treatment of ASC of the EGJ.

We report a case of ASC of the EGJ in which pathological findings indicated that multipotent undifferentiated carcinoma cells differentiated into both glandular and squamous cells. We also report a PR to monotherapy with S-1 in a patient with multiple postoperative metastases.

## Data Availability

Not applicable.
